# Variants in *RETN* gene are associated with steroid-induced osteonecrosis of the femoral head risk among Han Chinese people

**DOI:** 10.1186/s13018-020-1557-3

**Published:** 2020-03-06

**Authors:** Feimeng An, Litian Zhang, Hongyan Gao, Jiaqi Wang, Chang Liu, Ye Tian, Chao Ma, Jian Zhao, Kunzheng Wang, Jianzhong Wang

**Affiliations:** 1 Inner Mongolia Medical University, Hohhot, 010050 Inner Mongolia China; 2 Department of Trauma Orthopedics, The Second Affiliated Hospital of Inner Mongolia Medical University, Hohhot, 010030 Inner Mongolia China; 3 The Second Affiliated Hospital of Xi’an Jiaotong University, #157 Xi Wu Road, Xi’an, 710004 Shaanxi Province China

**Keywords:** Steroid-induced osteonecrosis of the femoral head (steroid-induced ONFH), Resistin (*RETN*), Genetic polymorphisms, Case-control study

## Abstract

**Background:**

Gene polymorphism has an important influence on *RETN* gene expression level, and the increased level of resistin encoded in *RETN* will lead to metabolic disorder, especially lipid metabolism. Moreover, steroid-induced osteonecrosis of the femoral head (steroid-induced ONFH) is closely related to lipid metabolism level, so this study is intended to explore the relationship of *RETN* polymorphisms with susceptibility to steroid-induced ONFH in the Chinese Han population.

**Methods:**

In this case-control study, eight single-nucleotide polymorphisms (SNPs) of *RETN* were genotyped by the Agena MassARRAY system in 199 steroid-induced ONFH patients and 200 healthy controls. The relationship between *RETN* polymorphisms and steroid-induced ONFH risk was assessed using genetic models and haplotype analyses. Odds ratio (OR) and 95% confidence intervals (CIs) were obtained by logistic regression adjusted for age.

**Results:**

We found significant differences in the distribution of HDL-C, TG/HDL-C, and LDL-C/HDL-C between the patients and the control group (*p* < 0.05). In allele model and genotype model analysis, rs34861192, rs3219175, rs3745368, and rs1477341 could reduce the risk of steroid-induced ONFH. Further stratified analysis showed that rs3745367 was related to the clinical stage of patients, and rs1477341 was significantly correlated with an increased TG level and a decreased TC/HDL-C level. The linkage analysis showed that two SNPs (rs34861192 and rs3219175) in *RETN* even significant linkage disequilibrium.

**Conclusions:**

Our results provide the firstly evidence that *RETN* gene polymorphisms were associated with a reduced risk of steroid-induced ONFH in Chinese Han population.

## Background

With the wide application of glucocorticoid in the treatment of rheumatic diseases, autoimmune diseases, hematopoietic system diseases, and other diseases, steroid-induced osteonecrosis of the femoral head (steroid-induced ONFH) has become the most common non-traumatic osteonecrosis of the femoral head (ONFH) type in clinical practice [1, 2]. Epidemiological studies in East Asia have shown that 47.4% of all cases diagnosed as non-traumatic ONFH arose as a direct result of steroids [3]. As a degenerative bone disease, it can cause the femoral head to collapse, which subsequently damage the hip joint and seriously reduces the patients quality of life and is difficult to reverse [3, 4]. However, it is challenging to fully elucidate the pathogenesis of steroid-induced ONFH due to the various effects of steroids on multiple systems involved in osteoblast differentiation, osteoblast and osteoclast apoptosis, lipid metabolism, calcium metabolism, and coagulation [5].

Previous studies have demonstrated that only a subset of patients develops ONFH within a few weeks of hormone therapy, suggesting that genetic factors may determine susceptibility to steroid-induced ONFH [6]. Single-nucleotide polymorphisms (SNPs) are the most frequent variation that occurs in a single nucleotide at a specific position in the genome. Numerous SNPs have been identified through sequencing, and many of them in critical genes such as *MMP*-*8* [1], *MMP*-*9* [7], *MMP-14* [8], *ABCB1* [9], and *VEGFA* [10] were demonstrated to be associated with steroid-induced ONFH susceptibility.

*RETN*, also known as *ADSF* and *FIZZ3*, is located on chromosome 19p13. *RETN* gene encodes resistin, which is a hormone secreted by fat cells and belongs to the cysteine-rich C-terminal domain proteins called resistin-like molecules [11]. It was first observed in adipocytes, then in monocytes, macrophages, and the spleen, and more importantly, in the human sebaceous glands and cultured sebaceous glands [12]. Some studies have demonstrated that elevated levels of resistin lead to metabolic disorders, which are associated with diabetes, non-insulin dependent, acquired systemic lipodystrophy, rheumatoid arthritis, etc. [13–15]. Most importantly, it is the product of the secretion of fat cells and its related pathways include lipogenesis [16]. Elevated levels of resistin may contribute to lipid metabolism disorders [15], which can lead to steroid-induced ONFH. Thus *RETN* may induce steroid-induced ONFH by affecting lipid metabolism.

To our knowledge, no previous studies have investigated the relationship between steroid-induced ONFH risk and *RETN* polymorphisms. Therefore, we conduct a case-control study to evaluate the possible relationship of *RETN* gene polymorphisms at allele, genotype, and haplotype interface with the development of steroid-induced ONFH among Chinese Han population.

## Materials and methods

### Study participants

The present hospital-based case-control study recruited 199 patients diagnosed with steroid-induced ONFH and 200 unrelated control subjects at the orthopedic hospital of Inner Mongolia Medical University (Inner Mongolia, China) from 2016 to 2019. These individuals were informed of the purpose of the study and signed informed consent.

We collected epidemiological information from standardized questionnaires and collected clinical information from medical records and imaging examinations to finally determine the case and control individuals. Patients with steroid-induced ONFH in this study had common clinical manifestations of hip pain, lower limb muscular atrophy, and joint dysfunction and were also receiving long-term steroid intake of more than 16 mg per day or high-dose impulsive treatment with steroids lasting more than 1 week prior to the onset of these symptoms [1]. An X-ray examination and additional magnetic resonance imaging (MRI) and bone scan analyses were performed on each patient as necessary. We also used strict exclusion criteria to exclude the following patients: patients with traumatic ONFH and other hip diseases; patients who consumed more than 400 ml of alcohol per week; patients with a history of severe disease or severe chronic disease, such as renal insufficiency, diabetes, and cardiovascular and cerebrovascular diseases. The included controls met the following criteria: they had no hip pain, anteroposterior and frog-leg lateral pelvic radiographs did not show any lesions, no history of thromboembolic events, no symptoms of hip disease, and no chronic diseases such as renal insufficiency, diabetes, and cardiovascular and cerebrovascular diseases.

### SNPs selection and genotyping

Individual demographics information and the clinical characteristics of patients were collected by well-trained interviewers. Subsequently, 5 mL of peripheral blood from each participant was collected by a specialized technician and stored into tubes containing ethylenediaminetetraacetic acid (EDTA). After centrifugation, the specimens were stored at − 80 °C until further analysis. All volunteers signed an informed consent form explaining the research purpose of our study. Genomic DNA was isolated from peripheral whole blood employing the Gold Mag-Mini whole blood genomic DNA purification kit (Gold Mag Co. Ltd., Xi’an, China) following the manufacturer’s instructions and quantified by a Nano Drop spectrophotometer 2000 C (Thermo Scientific, Waltham, MA, USA).

In this study, eight SNPs (rs7408174, rs34861192, rs34124816, rs3219175, rs3745367, rs3745368, rs3745369, rs1477341) were selected for genotyping. These SNPs had minor allele frequencies > 5% in the 1000 Genome Projects (http://www.internationalgenome.org/). Polymerase chain reaction (PCR) extension primers were designed for these SNPs by the MassARRAY Assay Design 3.0 software (Agena). SNP genotyping analysis was carried out at an Agena MassARRAY RS1000 Instrument (Shanghai, China) system according to the standard scheme recommended by the manufacturer, and data were managed and analyzed by the Agena Typer 4.0 software [17]. In addition, about 10% of the total samples were randomly selected to repeat genotyping, and the reproducibility was 100%.

### Statistical analyses

All of the statistical analyses were performed with the SPSS statistical package, version 19.0 (SPSS Inc., Chicago, IL, USA). Hardy–Weinberg equilibrium (HWE) of each SNP in the control group was tested by Fisher’s exact test. Allele frequencies and genotype frequencies for each SNP of case and control subjects were evaluated using the chi-squared test. Odds ratios (ORs) and 95% confidence intervals (CIs) were determined using logistic regression analysis with adjustments for age and gender. The wild-type allele was used as a reference. Multiple genetic model analyses (codominant, dominant, recessive, and log-additive) were applied using the PLINK software (http://pngu.mgh.harvard.edu/purcell/plink/) to assess the association between SNPs and steroid-induced ONFH. Finally, the Haploview software package (version 4.2) and the SHEsis software (http://analysis.bio-x.cn/myAnalysis.php) were used to analyze the pairwise linkage disequilibrium (LD), haplotype construction, and genetic association of polymorphisms. All *p* values in this study were two-sided, and *p* value of less than 0.05 was the cutoff value for statistical significance.

## Results

### The basic characteristics of study subjects

The clinical information and the demographic characteristics of both the cases and controls were shown in Table 1. No significant difference was observed between cases and controls in regard to age, gender, TC, TG, LDL-C, and TG/HDL-C. However, there were significant differences in the distribution of HDL-C, TG/HDL-C, and LDL-C/HDL-C between the two groups (*p* < 0.05).

**Table 1:** Characteristics of the participants

Variables	Cases (*n* = 199)	Controls (*n* = 200)	*p* value
Sex N			0.966^b^
Male	115	116	
Female	84	84	
Age, years (mean ± SD)	41.15 ± 12.934	41.20 ± 8.662	0.961^a^
Clinical stages			
Stage II	47		
Stage III	94		
Stage IV	58		
Hip lesions			
unilateral	59		
bilateral	140		
TC (mmol/L)	4.50 ± 0.923	4.54 ± 0.814	0.703
TG (mmol/L)	1.84 ± 1.455	1.76 ± 1.076	0.569
HDL-C (mmol/L)	1.07 ± 0.269	1.15 ± 0.214	0.000^a^
LDL-C (mmol/L)	2.64 ± 0.765	2.56 ± 0.728	0.285
TC/HDL-C	4.41 ± 1.178	4.04 ± 0.856	0.000^a^
TG/HDL-C	1.94 ± 1.930	1.64 ± 1.149	0.059
LDL-C/HDL-C	2.58 ± 0.821	2.30 ± 0.767	0.001^a^

*TC* total cholesterol, *TG* triglycerides, *LDL-C* low-density lipoprotein cholesterol, *LDL-C* high-density lipoprotein cholesterol

^a^*p* value was calculated by independent samples *t* test

^b^*p* value was calculated by chi-squared test

*p* < 0.05 indicates statistical significance

### The associations between *RETN* SNPs and steroid-induced ONFH

The basic information of the selected SNPs are presented in Table 2. Allele frequencies of all loci in controls were in accordance with Hardy–Weinberg equilibrium (*p* > 0.05). As indicated by the italicized *p* values in Table 2, the chi-squared test was used to assess the risk of genetic polymorphisms in the allele model, and it was found that rs34861192 (OR = 1.64, 95% CI = 0.44–0.93, *p* = 0.019), rs3219175 (OR = 0.61, 95% CI = 0.42–0.89, *p* = 0.010), rs3745368 (OR = 0.62, 95% CI = 0.41–0.93, *p* = 0.020), rs1477341 (OR = 0.74, 95% CI = 0.56–0.98, *p* = 0.036) were related to a reduced risk of steroid-induced ONFH.

**Table 2:** Basic information of candidate SNPs of *RETN* gene in this study

SNP ID	Gene	Band	Alleles A/B	MAF		HWE-*p*^a^	OR (95% CI)	*p*^b^ value
				Case	Control			
rs7408174	*RETN*	19	C/T	0.265	0.233	0.556	1.19(0.86–1.64)	0.287
rs34861192	*RETN*	19	A/G	0.136	0.198	0.654	*0.64(0.44–0.93)*	0.019*
rs34124816	*RETN*	19	A/C	0.075	0.100	1.000	0.73(0.45–1.20)	0.219
rs3219175	*RETN*	19	A/G	0.132	0.200	0.^659^	*0.61(0.42–0.89)*	0.010*
rs3745367	*RETN*	19	A/G	0.324	0.390	0.882	0.75(0.56–1.00)	0.052
rs3745368	*RETN*	19	A/G	0.113	0.171	1.000	*0.62(0.41–0.93)*	0.020*
rs3745369	*RETN*	19	C/G	0.303	0.337	0.636	0.85(0.63–1.15)	0.305
rs1477341	*RETN*	19	A/T	0.405	0.480	0.479	*0.74(0.56–0.98)*	0.036*

*SNP* single-nucleotide polymorphism, *HWE* Hardy–Weinberg equilibrium, *OR* odds ratio, *95% CI* 95% confidence interval, *MAF* minor allele frequency

*p*^a^ and *p*^b^ were calculated by chi-squared test

Italicized values are statistically significant

*p < 0.05 indicates statistical significance

### Associations between genotype frequencies and steroid-induced ONFH risk

As presented in Table 3, we further analyzed the relationship between genotype frequency and steroid-induced ONFH risk through multiple genetic models. We found that rs34861192 was associated with a lower steroid-induced ONFH risk in codominant (AA vs GG: OR = 0.10, 95% CI = 0.01–0.79, *p* = 0.0329), recessive (AA vs GG-AG: OR = 0.11, 95% CI = 0.01–0.85, *p* = 0.035), and log-additive models (OR = 0.63, 95% CI = 0.43–0.93, *p* = 0.019). Rs3219175 was identified to decrease the steroid-induced ONFH risk in codominant (AA vs GG: OR = 0.10, 95% CI = 0.01–0.79, *p* = 0.029), dominant (AG-AA vs GG: OR = 0.63, 95% CI = 0.41–0.98, *p* = 0.038), recessive (AA vs GG-AG: OR = 0.11, 95% CI = 0.01–0.86, *p* = 0.0360), and log-additive models (OR = 0.60, 95% CI = 0.41–0.88, *p* = 0.010). Rs3745368 was also related to decreasing steroid-induced ONFH risk in dominant (AG-AA vs GG: OR = 0.61, 95% CI = 0.38–0.95, *p* = 0.030), and log-additive models (A vs G: OR = 0.61, 95% CI = 0.41–0.92, *p* = 0.019). Rs1477341 also showed an association with the steroid-induced ONFH risk in dominant (AT-AA vs TT: OR = 0.62, 95% CI = 0.40–0.96, *p* = 0.03) and log-additive models (OR = 0.73, 95% CI = 0.55–0.98, *p* = 0.036).

**Table 3:** Analysis of the association between SNPs of RETN gene and steroid-induced ONFH risk

SNP ID	Model	Genotype	Control	Case	OR (95% CI)	*p* value
rs34861192	Codominant	GG	130	146	1	
		AG	61	52	0.76(0.49–1.18)	0.220
		AA	9	1	*0.10(0.01–0.79)*	0.029*
	Dominant	GG	130	146	1	
		AG-AA	70	53	0.67(0.44–1.03)	0.071
	Recessive	GG-AG	191	198	1	
		AA	9	1	*0.11(0.01–0.85)*	0.035*
	Log-additive	–	–	–	*0.63(0.43–0.93)*	0.019*
rs3219175	Codominant	GG	129	146	1	
		AG	62	50	0.71(0.46–1.11)	0.133
		AA	9	1	*0.10(0.01–0.79)*	0.029*
	Dominant	GG	129	146	1	
		AG-AA	71	51	*0.63(0.41–0.98)*	0.038*
	Recessive	GG-AG	191	196	1	
		AA	9	1	*0.11(0.01–0.86)*	0.036*
	Log-additive	–	–	–	*0.60(0.41–0.88)*	0.010*
rs3745368	Codominant	GG	137	156	1	
		AG	56	41	0.64(0.40–1.02)	0.059
		AA	6	2	0.29(0.06–1.45)	0.131
	Dominant	GG	137	156	1	
		AG-AA	62	43	*0.61(0.38–0.95)*	0.030*
	Recessive	GG-AG	193	197	1	
		AA	6	2	0.32(0.06–1.63)	0.170
	Log-additive	–	–	–	*0.61(0.41–0.92)*	0.019*
rs1477341	Codominant	TT	51	68	1	
		AT	105	90	0.64(0.41–1.02)	0.059
		AA	43	32	0.56(0.31–1.00)	0.051
	Dominant	TT	51	68	1	
		AT-AA	148	122	*0.62(0.40–0.96)*	0.030*
	Recessive	TT-AT	156	158	1	
		AA	43	32	0.74(0.44–1.23)	0.239
	Log-additive	–	–	–	*0.73(0.55–0.98)*	0.036*

*SNP* single-nucleotide polymorphism, *OR* odds ratio, *95% CI* 95% confidence interval

*p* value adjusted for age was calculated by logistic regression

Italicized values are statistically significant

*p < 0.05 indicates statistical significance

### Relationship between *RETN* SNPs and clinical features of steroid-induced ONFH

We also explored the relationship between the *RETN* SNPs and steroid-induced ONFH clinical features, including gender, hip lesions, clinical stages, and the expression level of lipid. We observed that rs3745367 shows association with the clinical stages in Table 4 (*p* = 0.049). In terms of lipid metabolism level of patients, it was found that compared with AA genotype carriers, HDL-C level of TT genotype carriers in rs1477341 was significantly higher, and TC level was lower. Meanwhile, the TC/HDL-C ratio of TT genotype carriers was significantly lower than that of AA genotype carriers (Table 5).

**Table 4:** The association of genotypes in *RETN* genes with the clinical phenotypes

SNP	Genotype	Gender		*p*	Hip lesions		*p*	Clinical stages			*p*
		Male	Female		Unilateral	Bilateral		Stage II	Stage III	Stage IV	
rs7408174	CC	8	6	0.775	5	9	0.285	3	5	6	0.694
	CT	42	35		18	59		19	39	19	
	TT	64	43		36	71		24	50	33	
rs34861192	AA	0	1	0.465	0	1	0.547	0	1	0	0.193
	AG	29	23		18	34		15	28	9	
	GG	86	60		41	105		32	65	49	
rs34124816	AA	94	77	0.105	51	120	0.650	41	78	52	0.557
	CA	20	6		8	18		6	14	6	
	CC	1	1		0	2		0	2	0	
rs3219175	AA	0	1	0.470	0	1	0.592	0	1	0	0.216
	AG	28	22		17	33		15	26	9	
	GG	86	60		41	105		31	66	49	
rs3745367	AA	12	9	0.775	3	18	0.263	3	14	4	0.049*
	AG	48	39		27	60		24	44	19	
	GG	55	36		29	62		20	36	35	
rs3745368	AA	1	1	0.308	0	2	0.650	0	2	0	0.613
	AG	28	13		12	29		9	21	11	
	GG	86	70		47	109		38	71	47	
rs3745369	CC	8	6	0.876	4	10	0.059	2	11	1	0.161
	CG	50	40		20	70		20	41	29	
	GG	54	37		35	56		24	41	26	
rs1477341	AA	24	8	0.063	9	23	0.809	6	21	5	0.090
	AT	48	42		25	65		21	43	26	
	TT	35	33		22	46		17	25	26	

*p* values were calculated by logistic regression

Italicized value is statistically significant

*p < 0.05 indicates statistical significance

**Table 5:** The association of genotypes in *RETN* genes with the clinical phenotypes

SNP	Genotype	TC (mmol/L)	TG (mmol/L)	HDL-C (mmol/L)	LDL-C (mmol/L)	TC/HDL-C	TG/HDL-C	LDL-C/HDL-C
rs34861192	AA(*n* = 1)	4.84	2.91	1.08	2.61	4.48	2.69	2.42
	AG(*n* = 52)	4.71 ± 0.93	1.97 ± 1.32	1.05 ± 0.27	2.75 ± 0.77	4.72 ± 2.48	2.13 ± 1.79	2.75 ± 0.86
	GG(*n* = 146)	4.43 ± 0.91	1.78 ± 1.50	1.07 ± 0.27	2.60 ± 0.76	4.30 ± 1.15	1.87 ± 1.98	2.52 ± 0.81
	*p*	0.156	0.551	0.073	0.587	0.090	0.653	0.670
rs3219175	AA (*n* = 1)	4.84	2.91	1.08	2.61	4.48	2.69	2.42
	AG (*n* = 50)	4.75 ± 0.93	2.01 ± 1.33	1.05 ± 0.28	2.77 ± 0.78	4.76 ± 1.23	2.17 ± 1.82	2.77 ± 0.87
	GG (*n* = 146)	4.42 ± 0.91	1.78 ± 1.50	1.07 ± 0.27	2.59 ± 0.76	4.29 ± 1.15	1.87 ± 1.98	2.51 ± 0.80
	*p*	0.097	0.492	0.835	0.356	0.058	0.587	0.152
rs3745368	AA (*n* = 2)	4.11 ± 0.64	1.13 ± 0.01	1.14 ± 0.23	2.20 ± 0.80	4.38 ± 1.67	1.18 ± 0.26	2.40 ± 1.37
	AG (*n* = 41)	4.37 ± 0.95	1.76 ± 1.61	1.04 ± 0.22	2.56 ± 0.81	4.35 ± 1.23	1.92 ± 2.24	2.55 ± 0.88
	GG (*n* = 156)	4.54 ± 0.91	1.87 ± 1.42	1.07 ± 0.28	2.66 ± 0.76	4.43 ± 1.17	1.96 ± 1.86	2.59 ± 0.80
	*p*	0.453	0.722	0.718	0.531	0.927	0.853	0.912
rs1477341	AA(*n* = 32)	2.00 ± 1.40	4.41 ± 0.82	1.01 ± 0.22	2.50 ± 0.71	4.52 ± 1.15	2.17 ± 1.69	2.56 ± 0.84
	AT(*n* = 90)	1.96 ± 1.47	4.57 ± 0.94	1.04 ± 0.25	2.71 ± 0.78	4.58 ± 1.16	2.10 ± 1.98	2.70 ± 0.79
	TT(*n* = 68)	1.62 ± 1.53	4.45 ± 0.97	1.13 ± 0.30	2.58 ± 0.78	4.12 ± 1.19	1.65 ± 2.06	2.39 ± 0.84
	*p*	0.302	0.588	0.049*	0.315	0.049*	0.292	0.066

*p* values were calculated by logistic regression

Italicized values are statistically significant

*p < 0.05 indicates statistical significance

### Associations between haplotype analyses and steroid-induced ONFH risk

**Fig. 1:**
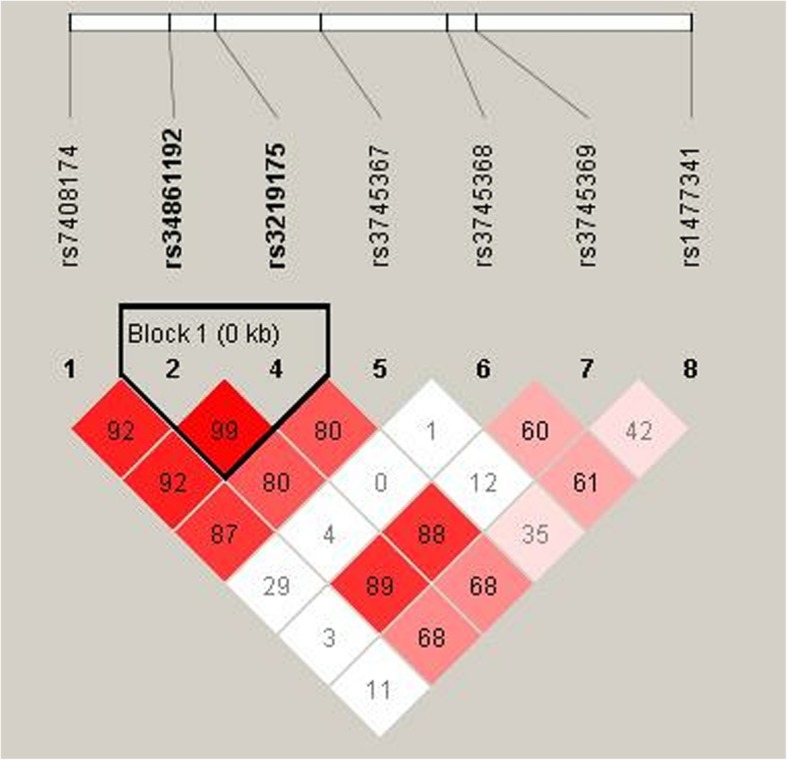
Haplotype block map for the eight SNPs in the *RETN* gene. Block 1 includes rs34861192 and rs3219175 with *D*’ = 1 (100%) for the corresponding variants

The linkage analysis showed that two SNPs (rs34861192 and rs3219175) in *RETN* exhibited significant linkage disequilibrium (Fig. 1).

### GTEx database analysis

Through GTEx database analysis, rs34861192 and rs3219175 (*p* = 4.00E-14, *p* = 1.60E-14) sites were observed to be associated with a reduced expression of *RETN* gene in whole blood.

## Discussion

It is well-known that genetic studies have provided insights into many diseases, including steroid-induced ONFH. This study was designed to investigate the contribution of genetic variation in *RETN* to steroid-induced ONFH risk in a Chinese Han population. Allele, genotype, and haplotype frequencies of eight SNPs in the *RETN* gene between steroid-induced ONFH patients and healthy controls were compared, and stratification analyses were conducted. Our study found that the rs34861192, rs3219175, rs3745368, and rs1477341 were linked to a reduced risk of steroid-induced ONFH. To our knowledge, this is the first research to clarify the correlation between *RETN* gene variants with the risk of steroid-induced ONFH in Chinese Han population.

The *RTEN* gene encodes a resistin, which is also considered to be a biomarker or mediator of metabolic and inflammatory diseases [18, 19]. Some studies have shown that elevated levels of serum resistin lead to metabolic disorders, including obesity, insulin resistance, type 2 diabetes, hypertension, dyslipidemia, and atherosclerotic cardiovascular disease [20–22]. Moreover, it has been shown that the plasma resistin concentration was largely determined by polymorphisms of *RETN*. For example, one study showed that plasma resistin was significantly correlated with rs34861192, rs34124816, rs3219175, rs3745367, and rs3745369 in *RETN* [23]. The study of Asano et al. found that rs34861192 and rs3745368 polymorphisms of *RETN* as robust and independent determinants of plasma resistin concentration [24]. An epigenome-wide association study suggested that rs34861192 and rs3219175 in the *RETN* promoter region may influence circulating resistin levels by affecting DNAm at cg02346997 and the abundance of *RETN* mRNA in monocytes [3]. Besides, the minor (A) allele of rs34861192 was also found to be associated with a lower plasma resistin level (*R*^2^ = 0.010) [24]. The GG genotype carriers of *RETN* rs3219175 and rs3481192 exhibited higher levels of log-resistin than A allele carriers [25]. After multiple testing corrections, the rs1477341 also had a strong correlation with resistin level [26]. In our study, we found that the frequency of the minor (A) allele of rs34861192, rs3219175, rs3745368, and rs1477341 was 0.136, 0.132, 0.113, and 0.405, respectively. All four SNPs were linked to a lower risk of steroid-induced ONFH. Therefore, we speculated that these SNPs may reduce the occurrence of metabolic disorder by inducing a lower level of resistin, ultimately reducing the risk of steroid-induced ONFH.

Moreover, the plasma resistin levels have been shown to be related to serum concentrations of HDL-cholesterol and triacylglycerol, IRI, and BMI [24]. In our study, we found that rs1477341 was significantly correlated with elevated HDL-C levels, and the HDL-C level of rs1477341-TT carriers was significantly higher than that of rs1477341-AA carriers. Besides, our study also found that rs1477341 can reduce the risk of steroid-induced ONFH. These results further suggested the correlation between rs1477341 and resistin levels, HDL-C levels, metabolic disorders, and osteonecrosis. As for linkage disequilibrium analysis, it was also found that rs34861192 and rs3219175 were all located in the same LD segment, and both of them have been reported to be closely related to circulating resistin levels [27]. Similarly, our study also found that rs34861192 and rs3219175 were located in the same LD block.

Of course, our research has some limitations. Firstly, the inherent selecting and information bias were the unavoidable problems because this is a hospital-based, single-center study. Secondly, the number of cases in our study is not large enough to rule out false-negative results, so a larger sample size is needed for further confirmation. Third, our current study is a foundation’s case-control study that requires further functional studies to understand the underlying genetic mechanism of steroid-induced ONFH. Despite the limitations noted above, our current findings provide scientific evidence for future studies on the effect of *RETN* on steroid-induced ONFH risk.

## Conclusions

To sum up, the present study confirmed for the first time that the *RETN* polymorphisms rs34861192, rs3219175, rs3745368, and rs1477341 were associated with a reduced risk of steroid-induced ONFH, which may provide the basis for elucidating the pathogenesis of osteonecrosis.

## Data Availability

The datasets used or analyzed during the current study are available from the corresponding author on reasonable request.

## Abbreviations

95% CI: 95% confidence intervals
HWE: Hardy–Weinberg equilibrium
LD: Linkage disequilibrium
MAF: Minor allele frequency
OR: Odds ratio
SNP: Single-nucleotide polymorphism
steroid-induced ONFH: Steroid-induced osteonecrosis of the femoral head
